# Genotyping of *Plasmodium falciparum* using antigenic polymorphic markers and to study anti-malarial drug resistance markers in malaria endemic areas of Bangladesh

**DOI:** 10.1186/1475-2875-11-386

**Published:** 2012-11-22

**Authors:** Jasmin Akter, Kamala Thriemer, Wasif A Khan, David J Sullivan, Harald Noedl, Rashidul Haque

**Affiliations:** 1 ICDDR,B, GPO Box 128, Dhaka, 1000, Bangladesh; 2 Department of Specific Prophylaxis and Tropical Medicine, Medical University of Vienna, Vienna, Austria; 3 Johns Hopkins Malaria Research Institute, Baltimore, MD, USA

**Keywords:** Malaria, Genotype, Anti-malarial drug resistance markers, *Plasmodium falciparum*, MSP-1 and pfcrt

## Abstract

**Background:**

In the past many regions of Bangladesh were hyperendemic for malaria. Malaria control in the 1960s to 1970s eliminated malaria from the plains but in the Chittagong Hill Tracts remained a difficult to control reservoir. The Chittagong Hill Tracts have areas with between 1 and 10% annual malaria rates, predominately 90-95% *Plasmodium falciparum*. In Southeast Asia, multiplicity of infection for hypo-endemic regions has been approximately 1.5. Few studies on the genetic diversity of *P. falciparum* have been performed in Bangladesh. Anderson *et al.* performed a study in Khagrachari, northern Chittagong Hill Tracts in 2002 on 203 patients and found that parasites had a multiplicity of infection of 1.3 by MSP-1, MSP-2 and GLURP genotyping. A total of 94% of the isolates had the K76T *Pfcrt* chloroquine resistant genotype, and 70% showed the N86Y *Pfmdr1* genotype. Antifolate drug resistant genotypes were high with 99% and 73% of parasites having two or more mutations at the *dhfr* or *dhps* loci.

**Methods:**

Nested and real-time polymerase chain reaction (PCR) methods were used to genotype *P. falciparum* using antigenic polymorphic markers and to study anti-malarial drug resistance markers in malaria endemic areas of Bangladesh.

**Results:**

The analysis of polymorphic and drug resistant genotype on 33 paired recrudescent infections after drug treatment in the period 2004 to 2008 in the Chittagong Hill Tracts, which is just prior to countrywide provision of artemisinin combination therapy. Overall the multiplicity of infection for MSP-1 was 2.7 with a slightly smaller parasite diversity post-treatment. The 13 monoclonal infections by both GLURP and MSP-1 were evenly divided between pre- and post-treatment. The MSP-1 MAD block was most frequent in 66 of the samples. The prevalence of the K76T PfCRT chloroquine resistant allele was approximately 82% of the samples, while the resistant Pfmdr1 N86Y was present in 33% of the samples. Interestingly, the post-treatment samples had a small but significantly higher frequency of the sensitive PfCRT alleles by RT-PCR.

**Conclusion:**

The parasite population retains high population diversity despite hypo-endemic transmission with retention, but decrease in the chloroquine-resistant allele and Pfmdr1 resistant alleles in the Chittagong Hill Tracts of Bangladesh.

## Background

Malaria is a major health problem in several Southeast Asian countries and is exacerbated by the development and spread of anti-malarial drug resistance. A recent report from WHO in 2009 described about 55,000 laboratory-confirmed malaria cases with more than 40 deaths per year in Bangladesh [1]. Estimated non-confirmed malaria cases are in the hundreds of thousands. The total annual mortality has decreased from 500 per annum the decade before. This may be due to the change to artemisinin-based combination therapy and greater use of malaria rapid diagnostic tests.

Resistance to chloroquine, the most widely used and affordable anti-malarial drug, had contributed to increased mortality and morbidity caused by *Plasmodium falciparum* infections during the 1990s in Bangladesh [2,3]. So far, very limited data are available on the efficacy of malaria treatments and the current situation of anti-malarial drug resistance in Bangladesh. In addition, only a few studies on the molecular characterization of the local parasite population have been performed. Anderson *et al.*[4] performed a study in Khagrachari, northern Chittagong Hill Tracts in 2002 on 203 patients and found that parasites had a multiplicity of infection of 1.3 by MSP-1, MSP-2 and GLURP genotyping. A total of 94% of the isolates had the K76T *Pfcrt* chloroquine resistant genotype, and 70% showed the N86Y *Pfmdr1* genotype. Antifolate drug resistant genotypes were high with 99% and 73% of parasites having two or more mutations at the *dhfr* or *dhps* loci [4].

It is very difficult to genotype malaria parasites accurately in areas of high transmission, where patients are often infected with multiple parasite strains. It is generally agreed that if any parasites in the pre-treatment sample persist after therapy, the subject is considered to have a recrudescence. However, when multiple parasite strains are present, the probability increases that at least one strain in the pre-treatment and recurrent samples may have the same genotype, leading to misclassification of a new infection as a recrudescence. Others have noted the importance of taking into account misclassification of new infections as recrudescence [5,6] but this has often been overlooked when interpreting genotyping-adjusted results from anti-malarial clinical trials [7]. Misclassification of a new infection as a recrudescence can be decreased by using multiple genotyping markers [8].

The purpose of the study was to genotype *P. falciparum* using antigenic polymorphic markers and to study anti-malarial drug resistance markers of the *P. falciparum* parasites in malaria endemic areas of Bangladesh. The study samples collected from three clinical trials conducted in malaria endemic areas of Bangladesh from 2004 to 2008. This study also provides a follow-up to the analysis performed in Khagrachari in 2002 [4] and also another analysis before countrywide artemisinin combination therapy began in 2007.

## Methods

### Study site and samples

Samples were collected from three clinical trials, studying anti-malarial drug efficacy, one conducted in Cox’s-Bazar district and another two in Banderban district, in the Chittagong Hill Tracts during 2004, 2005 and 2008 respectively. The study conducted in 2004 by Haque *et al.* on the efficacy of quinine plus sulphadoxine-pyrimethamine, in a situation where chloroquine was the first-line therapy, showed a treatment failure rate after PCR adjustment of 11.7% for Q+SP trial. In the year of 2005, artemether-lumefantrine was first-line therapy but, due to limited data on ACT efficacy in Bangladesh, Thriemer *et al.* conducted study on ACT efficacy and found a failure rate after PCR adjustment of 5.7% for ACT. Similarly, artemisinin-based combination (ACT) was first-line therapy in 2008 when a study conducted on the azithromycin plus artesunate showed a failure rate after PCR adjustment of 5.4% for the azithromycin-artesunate trial.

Samples were taken at Day 0 (pre-treatment) and at the day of recurrent parasitaemia (post-treatment) during 42 days’ follow-up. A total of 33 patients showed reappearance of parasites from the above-mentioned three studies and details of the studies were already published [9-11].

### Blood collection and microscopy

Venous blood samples were collected in an EDTA tube for microscopic examination and DNA extraction. Giemsa-stained blood films obtained from the patients were examined by light microscopy under an oil-immersion objective. Parasitaemia was estimated by counting the number of asexual forms of *P. falciparum* corresponding to 200 leukocytes in the thick blood film. The parasite density was calculated by assuming a leukocyte count of 8,000/μl blood [12]. Geometric mean parasite densities were not significantly higher (*p* >0.05) in patients who later showed reappearance of parasites than in patients who were classified as ACPRs and geomeans were comparable.

### Molecular analyse*s*

#### DNA extraction and nested PCR

Blood samples were stored at −20°C prior to DNA extraction. Genomic DNA was extracted from a total of 200 μl whole blood per patient using the Qiagen DNA extraction kit (QIAGEN, USA) according to manufacturer's instructions. Nested polymerase chain reaction (PCR) genotyping was performed both for the variable block 2 regions of merozoite surface protein-1 (MSP-1), and glutamate-rich protein (GLURP) genetic markers to assess potential multiplicity of *P. falciparum* infection [13]. Paired pre-treatment and post-treatment samples were analysed using parasite loci that exhibit repeat numbers of polymorphisms to distinguish true treatment failures from new infections.

Briefly, block 2 of MSP-1 and region II of GLURP were amplified by two rounds of PCR using primers and amplification conditions previously described by Snounou and others [14]. Ten microlitres of the PCR products were resolved by electrophoresis on a 2% agarose gel and sized against 50-basepair (bp) molecular weight marker (New England Biolabs, Beverly, MA, USA).

The banding pattern of the post-treatment parasites was compared with matched pre-treatment samples. Post-treatment and primary infection parasites showing identical bands were considered a true treatment failure, while non-identity indicated a new infection.

Point mutations in the *P. falciparum* chloroquine resistance transporter (*Pfcrt*) gene and in the *P. falciparum* multidrug resistance 1 gene (*Pfmdr1*) were analysed by PCR-RFLP methods as described previously [15,16]. Multiplex-real-time PCR was performed and compared with the PCR-RFLP result for K76T in pre-treatment and post-treatment samples.

### Real-time PCR and *pfcrt* haplotype analysis

Multiplex real-time PCR was performed using a BioRad CFX 96 Multicolor Real time Detection System thermocyler (Bio-Rad, Hercules, CA). The reaction used IQ powermix (BioRad) with 0.2 μM primer and probes, 1 μl DNA for controls and 2 μl blood DNA for sample in a final volume of 10 μl. All reactions were run in duplicate. Real-time PCR cycling conditions consisted of an initial denaturation step (5 min at 95°C), followed by 40 amplification cycles (10 sec at 95°C; 30 sec at 52°C; 30 sec at 65°C). 3D7 and W2 strains were used as positive controls for K76 and 76T respectively. Primers and probes were designed to detect K76T mutation associated chloroquine (CQ) resistance by TagMan multiplex real-time PCR assay. Probes are crt76-CVMNK (CQ sensitive), crt76-CVIET (CQ resistant) in Southeast Asia and Africa (Old World) [17]. Probes were also designed to bind separately with the sensitive and resistant type and data were analysed on the basis of *C*t value (threshold cycle) and in relation to the relative fluorescence unit (RFU) for the respective haplotype. The commonly used *C*t value (threshold cycle) was calculated as the intersection of a line drawn through the baseline and a line drawn through the rise line. *C*t estimates made using this method were found to be more accurate and consistent than those reported by the instrument control software. A cut-off for RFU was determined by endpoint analysis in accordance with the manufacturer’s instructions. The sequence of primers and probes is given in Table 1.

**Table 1:** Oligonucleotides used in this study (listed 5′ to 3′)

**Name**	**Sequence**
pfcrt F	5′-TGT GCT CAT GTG TTT AAA CTT-3′
pfcrt R	5′-GGA TGT TAC AAA ACT ATA GTT ACC-3′
pfcrt 76-CVMNK	Texas- TGT GTA ATG AAT AAA ATT TTT GCT AA
pfcrt 76-CVIET	FAM- TGT GTA ATT GAA ACA ATT TTT GCT AA

## Results

### Genotyping using MSP-1 and GLURP markers

**Figure 1:**
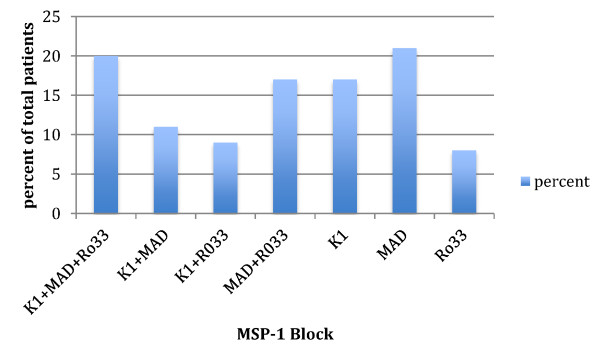
**Distribution of merozoite surface protein-1 (*msp-1*) polymorphic loci of *Plasmodium falciparum *from patient isolates.**

**Figure 2:**
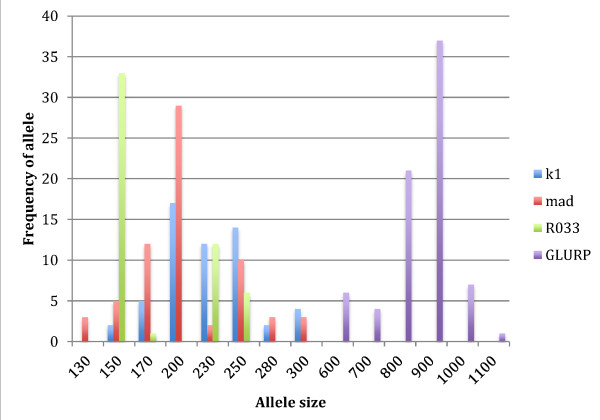
**Alllelic distribution of the merozoite surface protein-1 (*msp-1*) and glutamate-rich protein (*glurp*) of *Plasmodium falciparum *from patient isolates.**

A total of 33 sample pairs were collected from patients who failed anti-malarial drug treatment and were successfully analysed for genetic diversity using *msp-1* and *glurp* loci. Amplification products of the MSP-1 allelic family K1 was positive in 36 (54%) of the 66 samples, and yielded six different allele fragments (150–280 bp) with a predominate 200 bp allele (Table 2 and Figure 1). The MSP-1 allelic family MAD20 was detected in 44 (66%) samples and produced eight different allele fragments (130–300 bp) with a predominant 200 bp allele. The *msp-1* RO33 allele was detected in 34 (52%) samples, and produced amplification products of four different sizes with a predominant 150 bp fragment. Within MSP-1 allelic families MAD 20 showed higher frequency of PCR positivity and alleles than K1 and RO33 in total samples. The region II of *glurp* was detected in all of the samples and produced six different fragments (600–1,100 bp) with a predominant 900 bp allele (Figure 2). MSP-1 was more diverse than *glurp −*1 with 192 possible alleles versus 6, when the specific loci of these genes that were assessed. GLURP indicated monoclonal infections in 83% of the samples partially because of the frequency of the 900 bp allele of *glurp* at close to half of genotypes 37/76 (49%), while *msp-1* indicated monoclonal in only 20%. The frequency of the predominant *msp-1* alleles were −17/56 (30%) for K1, 29/67 (43%) for MAD-20 and 33/52 (63%) for Ro33. K1 and MAD had more normal distribution of allele numbers while Ro33 and *glurp* had predominance of single allele frequency. The most common genotype was K1-200 bp and *glurp −*900 bp, which occurred in five of 66 samples. This would equate to at most a 8% chance of finding a similar genotype in pre- and post-treatment samples given a re-infection rather than a recrudescent infection. The multiplicity of infection was 2.7 for all three *msp-1* blocks together and 1.5-1.6 individually, while *glurp* had a similar MOI of 1.2 to the 2 allele RFLP of *Pfcrt* and *Pfmdr1* (Table 2).

**Table 2:** **Parasite genotypes based on polymerase chain reaction (PCR) products of isolates of *Plasmodium falciparum *obtained before treatment from 33 patients who failed treatment with anti-malarial drugs in a malaria-endemic region of Southeastern Bangladesh**

**Loci**	**Number of alleles**	**Number positive**	**Number MOI≥2**	**MOI Pre TX**	**number positive**	**Number MOI≥2**	**MOI Post TX**	**MOI both**
MSP-1 total		33	27	3.0	33	26	2.3	2.7
K1	6	19	10	1.5	17	10	1.6	1.6
MAD	8	25	12	1.5	19	11	1.6	1.5
R033	4	20	13	1.6	13	7	1.5	1.6
GLURP	6	32*	6	1.2	33	5	1.2	1.2
CRT-RFLP	2	33	6	1.2	33	3	1.1	1.1
MDR1	2	33	10	1.3	33	3	1.1	1.2

* Note: one of paired samples in baseline has not amplified for GLURP.

### Complexity of genotypes in pre- and post-treatment samples

Analysis of the genetic diversity in 33 paired isolates using *msp-1* and *glurp* showed that *msp-1* had a higher MOI of 3.0 pre-treatment *versus* 2.3 post-treatment. The individual *msp-1* blocks and *glurp* all had similar pre- or post-treatment MOI. Pre-treatment 27 (81%) and six (18%) of the 33 samples were polymorphic for *msp-1* or *glurp*, respectively, which was similar to post-treatment MOI (Table 2). There were three (5%) samples with a maximum of six genotypes using each of the MSP-1 blocks.

Persistent of identical alleles for GLURP pre- or post-treatment occurred in 22/33 samples and in 20 of 33 MSP-1, while in both in 14 of 33. Four paired samples were completely identical with even more than one *msp-1* alleles present at both times. Five of 33 pairs had completely different alleles for both *msp-1* and *glurp*. Novel allele present in 13 cases of the post-treatment samples that was not present in the pre-treatment sample and loss of one or more alleles following treatment in 11 cases of the post-treatment samples.

### Prevalence of *Plasmodium falciparum* drug resistance markers

Molecular analysis of anti-malarial drug resistance markers, such as *Pfcrt* and *Pfmdr-1,* by PCR-restriction fragment length polymorphisms (RFLP) in pre-treatment and post-treatment samples showed a high prevalence of mutant *Pfcrt* K76T 54/66 (82%) and a low prevalence of mutant *Pfmdr1* N86Y 25/66 (33%) and mutant Y184F 8/66 (12%). Among the 33 paired samples, nine and 13 were heterozygous for *Pfcrt* and *Pfmdr1* (Table 3). By RFLP the post-treatment prevalence of only sensitive *Pfcrt* was doubled from four to eight while any sensitive were 10 to 11.

**Table 3:** Classification by PCR genotyping method of post-treatment isolates

**Parasites genotypes**	**PfCRT K76T RFLP**		**PfCRT K76T RTPCR**		**MDR1 N86Y RFLP**		**MDR1 Y184F RFLP**	
	**Pre Tx**	**Post Tx**	**Pre Tx**	**Post Tx**	**Pre Tx**	**Post Tx**	**Pre Tx**	**Post Tx**
Sensitive	4	8	1	9	18	23	31	27
Sensitive and resistant	6	3	5	1	10	3	1	1
Resistant	23	21	27	23	5	7	1	5

The *Pfcrt* RT-PCR agreed with post-treatment RFLP with more variance on the pre-treatment alleles. The discordance was RFLP overcalling or undercalling combined sensitive and resistant alleles in the sample six of eight times.

The wild type *Pfmdr1* alleles at position 86 and 184 was present in 36/66 of the samples, an additional six had wild type N86 with resistant Y184F and 22 samples had resistant N86Y with sensitive Y184. Only two samples were resistant at both amino acids. The double sensitive allele and the 184 resistant allele have been associated with more chloroquine sensitivity, but more resistance to mefloquine or artemisinin derivatives [18].

## Discussion

The findings on genetic diversity based on the polymorphic genes *msp-1* and *glurp* of *P. falciparum* isolates from a malaria-endemic region of Bangladesh, complementing earlier work in a different location [1,8]. Multiplicity of infection with MSP-1 is slightly higher. The *glurp* RFLP did not add much to genotyping with lower frequency of diverse alleles. The post-treatment MOI was slightly less than pre-treatment, this may be due to clearance of sensitive strains through following treatment. The somewhat higher population diversity is surprising for the level of endemic malaria but may reflect recent larger diversity with meso-endemic malaria.

The chloroquine-resistant allele appears to be decreasing slightly in frequency just before the advent of widespread use of the artemisinin derivatives. The high population diversity with sensitive *Pfcrt* may translate into return of the chloroquine-sensitive genotype given that the resistant genotype does not appear to be fixed in the population.

Despite a slight discordance between RFLP and RT-PCR for the *Pfcrt* alleles the greater sensitivity of detection and ease of use with an existent RT-PCR machine makes the RT-PCR preferable.

This study shows the diversity and complexity of the *P. falciparum* population in malaria endemic areas of Bangladesh prior to deployment of country-wide artemisinin combination therapy. The approach used in this study could be used in addition to other molecular methods as a part of a surveillance programme for monitoring drug resistance patterns in malaria infections.

## Conclusion

The parasite population retains a high population diversity despite hypo-endemic transmission with retention, but decrease in the frequency of the chloroquine-resistant allele and *Pfmdr1* resistant alleles.

## Competing interests

The authors declare that they have no competing interests.

## Authors’ contributions

JA designed the study and performed laboratory work, including data analysis and manuscript preparation. KT, WA and HN directed the field studies from where samples were collected. DJS made substantial correction of manuscript with analysis and interpretation of the data. RH was involved at all stages of the study and lead the manuscript preparation. All authors read and approved the final manuscript.

## References

[B1] World Health Organization 2005 http://rbm.who.int/wmr2005/profiles/bangladesh.pdf

[B2] Mayor AG Gomez-Olive X Aponte JJ Casimiro S Mabunda S Dgedge M Barreto A Alonso PL Prevalence of the K76T mutation in the putative Plasmodium falciparum chloroquine resistance transporter (pfcrt) gene and its relation to chloroquine resistance in Mozambique J Infect Dis 2001 183 1413 1416 10.1086/319856 11294676

[B3] Trape JF Pison G Preziosi MP Enel C Desgrees du Lou A Delaunay V Samb B Lagarde E Molez JF Simondon F Impact of chloroquine resistance on malaria mortality C R Acad Sci 1998 321 689 697 10.1016/S0764-4469(98)80009-7 9769862

[B4] van den Broek IV van der Wardt S Talukder L Chakma S Brockman A Nair S Anderson TC Drug resistance in Plasmodium falciparum from the Chittagong Hill Tracts. Bangladesh Trop Med Int Health 2004 9 680 687 10.1111/j.1365-3156.2004.01249.x 15189458

[B5] Brockman A Paul RE Anderson TJ Hackford I Phaiphun L Looareesuwan S Nosten F Day KP Application of genetic markers to the identification of recrudescent Plasmodium falciparum infections on the northwestern border of Thailand AmJTrop Med Hyg 1999 60 14 21 10.4269/ajtmh.1999.60.14 9988316

[B6] Guthmann JP Pinoges L Checchi F Cousens S Balkan S van Herp M Legros D Olliaro P Methodological issues in the assessment of antimalarial drug treatment: analysis of 13 studies in eight African countries from 2001 to 2004 Antimicrob Agents Chemother 2006 50 11 3734 3739 10.1128/AAC.01618-05 16954313 PMC1635238

[B7] Greenhouse B Dokomajilar C Hubbard A Rosenthal PJ Dorsey G Impact of transmission intensity on the accuracy of genotyping to distinguish recrudescence from new infection in antimalarial clinical trials Antimicrob Agents Chemother 2007 51 3096 3103 10.1128/AAC.00159-07 17591848 PMC2043236

[B8] Snounou G Beck HP The use of PCR genotyping in the assessment of recrudescence or reinfection after antimalarial drug treatment Parasitol Today 1998 14 462 467 10.1016/S0169-4758(98)01340-4 17040849

[B9] Haque R Thriemer K Wang Z Sato K Wagatsuma Y Salam MA Akther S Akter J Fukuda M Miller RS Noedl H Therapeutic efficacy of artemether-lumefantrine for the treatment of uncomplicated Plasmodium falciparum malaria in Bangladesh AmJTrop Med Hyg 2007 76 39 41 17255226

[B10] Thriemer K Starzengruber P Khan WA Haque R Marma AS Ley B Vossen MG Swoboda P Akter J Noedl H Azithromycin combination therapy for the treatment of uncomplicated falciparum malaria in Bangladesh: an open-label randomized, controlled clinical trial J Infect Dis 2010 202 392 398 10.1086/653710 20557237

[B11] Thriemer K Haque R Wagatsuma Y Salam MA Akther S Attlmayr B Fukuda M Schaecher K Miller RS Noedl H Therapeutic efficacy of quinine plus sulfadoxine-pyremethamine for the treatment of uncomplicated falciparum malaria in Bangladesh AmJTrop Med Hyg 2006 75 645 649 17038687

[B12] Moody A Rapid diagnostic tests for malaria parasites Clin Microbiol Rev 2002 15 1 66 78 10.1128/CMR.15.1.66-78.2002 11781267 PMC118060

[B13] Farnert A Arez AP Babiker HA Beck HP Benito A Bjorkman A Bruce MC Conway DJ Day KP Henning L Genotyping of Plasmodium falciparum infections by PCR: a comparative multicentre study Trans R Soc Trop Med Hyg 2001 95 225 232 10.1016/S0035-9203(01)90175-0 11355566

[B14] Snounou G Zhu X Siripoon N Jarra W Thaithong S Brown KN Viriyakosol S Biased distribution of msp1 and msp2 allelic variants in Plasmodium falciparum populations in Thailand Trans R Soc Trop Med Hyg 1999 93 369 374 10.1016/S0035-9203(99)90120-7 10674079

[B15] Djimde A Doumbo OK Cortese JF Kayentao K Doumbo S Diourte Y Dicko A Su XZ Nomura T Fidock DA A molecular marker for chloroquine-resistant falciparum malaria N Engl J Med 2001 344 257 263 10.1056/NEJM200101253440403 11172152

[B16] Duraisingh MT Jones P Sambou I von Seidlein L Pinder M Warhurst DC The tyrosine-86 allele of the pfmdr1 gene of Plasmodium falciparum is associated with increased sensitivity to the anti-malarials mefloquine and artemisinin Mol Biochem Parasitol 2000 108 13 23 10.1016/S0166-6851(00)00201-2 10802315

[B17] Gadalla NB Elzaki SE Mukhtar E Warhurst DC El-Sayed B Sutherland CJ Dynamics of pfcrt alleles CVMNK and CVIET in chloroquine-treated Sudanese patients infected with Plasmodium falciparum Malar J 2010 9 74 10.1186/1475-2875-9-74 20226032 PMC2848148

[B18] Pickard AL Wongsrichanalai C Purfield A Kamwendo D Emery K Zalewski C Kawamoto F Miller RS Meshnick SR Resistance to antimalarials in Southeast Asia and genetic polymorphisms in pfmdr1 Antimicrob Agents Chemother 2003 47 2418 2423 10.1128/AAC.47.8.2418-2423.2003 12878499 PMC166057

